# Ultracompact and high efficient silicon-based polarization splitter-rotator using a partially-etched subwavelength grating coupler

**DOI:** 10.1038/srep27949

**Published:** 2016-06-16

**Authors:** Yin Xu, Jinbiao Xiao

**Affiliations:** 1 National Research Center for Optical Sensing/Communications Integrated Networking, School of Electronic Science and Engineering, Southeast University, Nanjing 210096, China

## Abstract

On-chip polarization manipulation is pivotal for silicon-on-insulator material platform to realize polarization-transparent circuits and polarization-division-multiplexing transmissions, where polarization splitters and rotators are fundamental components. In this work, we propose an ultracompact and high efficient silicon-based polarization splitter-rotator (PSR) using a partially-etched subwavelength grating (SWG) coupler. The proposed PSR consists of a taper-integrated SWG coupler combined with a partially-etched waveguide between the input and output strip waveguides to make the input transverse-electric (TE) mode couple and convert to the output transverse-magnetic (TM) mode at the cross port while the input TM mode confine well in the strip waveguide during propagation and directly output from the bar port with nearly neglected coupling. Moreover, to better separate input polarizations, an additional tapered waveguide extended from the partially-etched waveguide is also added. From results, an ultracompact PSR of only 8.2 μm in length is achieved, which is so far the reported shortest one. The polarization conversion loss and efficiency are 0.12 dB and 98.52%, respectively, together with the crosstalk and reflection loss of −31.41/−22.43 dB and −34.74/−33.13 dB for input TE/TM mode at wavelength of 1.55 μm. These attributes make the present device suitable for constructing on-chip compact photonic integrated circuits with polarization-independence.

Silicon-on-insulator (SOI), a prominent platform for silicon photonics, benefited from high index contrast and well-established complementary metal-oxide-semiconductor (CMOS) compatible processing has attracted considerable interest for building compact and high efficient photonic integrated circuits (PICs) including components, modules, and systems to realize the on-chip optical interconnect with high density and low power consumption^1^,^2^,^3^. However, major challenges still exist, particularly for the strong polarization dependence of SOI functional devices due to high index contrast of the SOI materials, which will limit PICs in the SOI platform from large-scale commercialization in optical communications^4^. To overcome this drawback, polarization diversity scheme composed of polarization splitters and rotators is normally leveraged to realize a polarization-transparent circuit^5^. In such a scheme, the key constituent elements, e.g., polarization splitters^6^,^7^ and rotators^8^,^9^, are usually separate components so that the length of the whole device is very long. Apparently, it is against the dense integration with high reliability and stability. Accordingly, a more suitable choice is to use polarization splitter-rotators (PSRs) that can complete the functionalities of polarization splitting and rotating simultaneously only in one device. Over the years, great efforts have been made towards PSRs by using various kinds of structures, e.g., asymmetrical directional couplers (ADCs)^10^,^11^, bi-layer tapers^12^,^13^, Y-junctions^14^, multimode interference (MMI) couplers^15^,^16^, and their mechanisms can be roughly categorized into two types: mode coupling and mode evolution. For the former type, ADCs are usually the most commonly used structure where precise phase matching condition determined by the device geometry and operating wavelength should be satisfied, thus they are inherently fabrication sensitive and wavelength dependent. To effectively compensate fabrication errors, tapered^17^ and taper-etched DCs^18^ have been proposed, but at the expense of relatively large footprints (>100 μm^17^ and >120 μm^18^). For the latter type, adiabatic transition is employed, which has better fabrication tolerance and larger bandwidth, and a typical mode evolution-based PSR is composed of a TM_0_-to-TE_1_ polarization rotator and a cascaded TE_1_-to-TE_0_ mode-order converter^10^,^12^,^13^,^14^,^15^,^16^. For instance, the TM_0_-to-TE_1_ polarization rotator is mainly realized by using bi-layer tapers^12^,^13^, different upper-cladding materials^10^,^16^, or rib waveguides^19^ to break the vertical symmetry, and the TE_1_-to-TE_0_ mode-order converter is mostly achieved by using ADCs^10^, adiabatic couplers^12^, mode-sorting Y-junctions^14^, MMI couplers^15^,^16^ or Mach-Zehnder interferometers^13^. Thus, the total device length inevitably needs to be very long, e.g., ~100 μm^10^, 475 μm^12^, 96 μm^14^, ~200 μm^15^, 190 μm^16^ and ~120 μm^13^, to achieve reasonable performance, greatly limiting the integration density on-chip. Moreover, PSRs based on silicon nitride (Si_3_N_4_)-on-SOI platform^20^, operated at bi-wavelength (1310 nm and 1550 nm)^21^, 300 nm broadband around wavelength of 1550 nm^22^, or mid-infrared range^23^ have also been proposed, respectively, but the obtained device lengths are still quite long (576 μm^20^, 138 μm^21^, 300 μm^22^, and ~470 μm^23^). Therefore, it is greatly required to explore new approaches to effectively reduce the dimensions of PSRs to fulfill the requirements of dense integration on-chip with polarization-independence.

Recently, subwavelength gratins (SWGs), a grating pitch substantially smaller than the wavelength of light in the structure, behaving as a homogeneous media without diffraction effect, offer a new degree of freedom for the design of novel photonic devices since the effective index of the waveguide core can be easily engineered by changing the duty cycle^24^,^25^. Based upon this unique feature, SWGs have been applied into a variety of photonic devices with enhanced performance, e.g., fiber-chip grating couplers^26^, wavelength independent MMI couplers^27^, waveguide crossings^28^, power splitters^29^, and other fundamental building blocks (SWG-based tapers^30^,^31^, bends^32^, DCs^32^, and microring resonators^33^). Moreover, SWGs may also be employed to design compact and high efficient PSRs because the key features, such as structural birefringence and wavelength dependence of the device, can be adjusted more flexibly and efficiently using their effective index engineered structures. For instance, Xiong *et al*. proposed a PSR which comprises a silicon wire waveguide coupled to a SWG waveguide in an ADC with improved fabrication tolerance and the TM-to-TE polarization conversion loss (PCL) was 0.13 dB at wavelength of 1.55 μm^34^. However, its bandwidth was intrinsically limited by the ADC structure and the device footprint was relatively large (~50 μm for the total length). Consequently, ultracompact and broadband schemes for PSRs with reasonable performance are still needed to be proposed and optimized for on-chip polarization-transparent circuits or polarization-division-multiplexing transmission systems.

In this paper, we propose an ultracompact and high efficient silicon-based PSR based on a partially-etched SWG coupler between the input and output waveguides, where SWG-tapered transitions are combined at both ends. The dimensions of the SWG coupler and its lateral partially-etched waveguide including SWG-tapered transitions are optimized to make the input TE mode couple to the adjacent partially-etched waveguide gradually and convert to the output TM mode simultaneously at the cross port, while the input TM mode is output from the bar port with nearly neglected coupling. Meanwhile, an additional tapered waveguide extended from the partially-etched waveguide is added in the lateral end of the input SWG-tapered transition to better separate input polarizations. Compared with PSRs reported earlier, the present one has the shortest size since the input TE mode is directly coupled and converted to the output TM mode without some intermediate modes such as TE_1_ mode as a bridge used in many previous schemes^10^,^12^,^13^,^14^,^15^,^16^,^17^,^20^,^21^,^22^,^23^, and the coupling and converting processes are reasonably combined together instead of using cascaded parts composed of separate splitters and rotators. From results, the obtained device length is only 8.2 μm, which is about one order of magnitude smaller than that of previous reports^10^,^13^,^14^,^15^,^16^,^17^,^18^,^21^ and also quite smaller than that of SWG-based PSR assisted by an ADC structure^34^, more details are summarized in Fig. 1(a). Meanwhile, PCL and crosstalk (CT) of the input TE mode are 0.12 dB and <−31 dB, which are also superior to those of previous simulation works^14^,^15^,^19^,^21^,^22^,^34^, and insertion loss (IL) and CT of the input TM mode are 0.31 dB and <−22 dB, respectively, almost without significant deterioration, including the bandwidth of ~104 nm for keeping CT < −20 dB. To our knowledge, this is the first PSR reported so far whose footprint is less than 10 μm with reasonable performance.

## Results

### Device structure and principle

**Figure 1:**
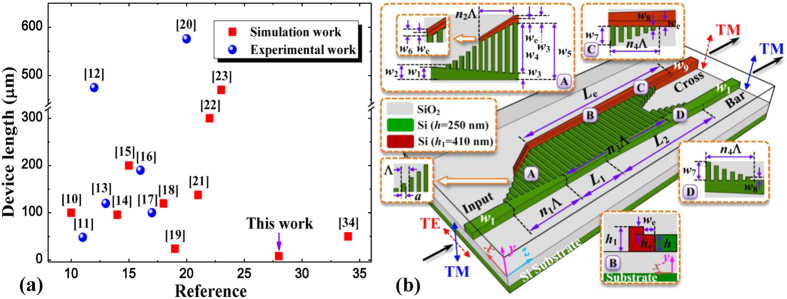
Review of the PSR and schematic of the proposed device. (**a**) Summary of the device length of the reported PSRs. (**b**) Schematic layout of the proposed PSR based on partially-etched SWG coupler, where its enlarged views are also illustrated (A: input SWG-tapered transition, B: partially-etched waveguide, C/D: output SWG-tapered transition at the cross/bar port). *n*_1_~ *n*_4_: SWG period numbers, *w*_1_, *w*_3_, *w*_6_, *w*_9_: strip waveguide width, *w*_2_, *w*_4_, *w*_5_, *w*_7_, *w*_8_: SWG waveguide width, *w*_e_ (*L*_e_): etching width (length), *L*_1_: strip wavelength length with a uniform with, *L*_2_: output tapered strip waveguide length, *a*: high index segment width in SWG, Λ: SWG pitch width.

Figure 1(b) shows the three-dimensional (3D) schematic of our proposed PSR together with enlarged views of the input/output SWG-tapered transitions and cross-section of the partially-etched waveguide region, where SWG coupler has the same pitch width and duty cycle of Λ, *a*/Λ (*a* is the width of high index segment), respectively. For the input TE mode, it is firstly tending to the SWG region as it enters into the input SWG-tapered transition with its width being tapered from *w*_2_ to *w*_5_ in a period number of *n*_1_ since it cannot be well-supported by the input strip waveguide with its width being tapered from *w*_1_ to *w*_3_. And then, it is further coupled to the adjacent partially-etched waveguide through the SWG multimode waveguide with its width of *w*_4_ in a period number of *n*_3_, where the mode conversion from coupled TE mode to output TM mode is also taken place in here using an asymmetrical structure created by the partially-etched waveguide. Finally, the converted TM mode outputs from the cross port, where the output SWG-tapered transition is employed to reduce the coupling loss with the SWG width being tapered from *w*_7_ to *w*_8_ in a period number of *n*_4_. To efficiently enhance the device performance, we also introduce an additional tapered waveguide with its width being tapered from *w*_3_ to *w*_6_ in a length of *n*_2_Λ in the lateral end of the input SWG-wire tapered waveguide, where its etching structure is similar with that of the partially-etched waveguide. As to the input TM mode, it always transmits along the strip waveguide and directly outputs from the bar port nearly neglecting the existence of the partially-etched SWG coupler because the chosen dimensions of the strip waveguide can confine it well for propagation.

If not specified, the computational parameters under the following analysis are as follows: refractive indices of the high- and low-index regions are, respectively, taken as 3.478 (Si) and 1.444 (SiO_2_) at wavelength of 1.55 μm^35^. The partially-etched waveguide has a full waveguide thickness of *h*_1_ (=410 nm), an etching depth of *h*_e_ (=160 nm) with etching length and width of *L*_e_ and *w*_e_, respectively, and other waveguide regions have the same thickness of *h* (=250 nm). In addition, the width of the input/output waveguide is set to be *w*_1_ = 400 nm to ensure the single-mode operation except for the output waveguide at the cross port whose width is set to be *w*_9_ = 350 nm due to its increased waveguide thickness. The structural and material parameters are also labeled in Fig. 1(b).

### Waveguide modal analysis

**Figure 2:**
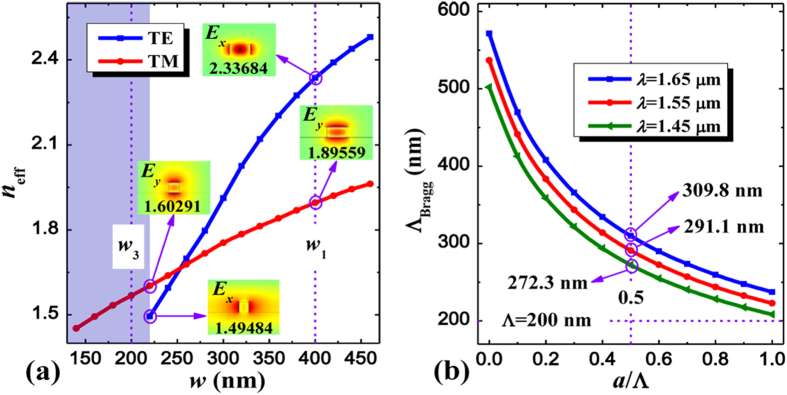
Modal analysis and SWG period width. (**a**) Effective indices *n*_eff_ of guided-modes for the individual strip waveguide as its width *w* varies, where the insets show the major component of electric field distribution of both polarizations at the widths of 400 nm and 220 nm, respectively., and *w*_1_, *w*_3_ are set to be 400 nm and 200 nm. The shadow region represents the TE mode is cutoff. (**b**) Bragg period of the grating Λ_Bragg_ as a function of its duty cycle *a*/Λ for different wavelengths, where SWG period width and its duty cycle are chosen to be 200 nm and 0.5 in order to effectively suppress the diffraction effect and alleviate the fabrication requirements.

For the proposed PSR based on SOI platform, the modal characteristics of silicon-based strip waveguides should be analyzed in order to determine their suitable dimensions to efficiently separate input polarizations within the input SWG-tapered transition. To do this, a full-vectorial mode solver based on the finite-difference frequency-domain (FDFD) method is utilized^36^. Figure 2(a) illustrates the calculated effective indices *n*_eff_ of guided-modes for the individual strip waveguide as its width varies for both polarizations. Obviously dissimilar modal features can be observed and the TE mode is tending to cutoff whereas the corresponding TM mode can still confine relatively well within the waveguide if its width is less than 220 nm, which offers a way to separate input polarizations. Accordingly, we set *w*_3_ to be 200 nm, which is connected with the input waveguide (*w*_1_ = 400 nm) using a waveguide taper, and the SWG coupler is employed at the side of this waveguide taper to efficiently guide this cutoff TE mode to the adjacent waveguide while the propagation of TM mode is nearly unaffected, thus leading to the separation of input polarizations. It is noted that the SWG coupler plays a pivotal role in this structure and its properties should also be detailedly analyzed. Figure 2(b) shows the Bragg period (Λ_Bragg_) of the grating as its duty cycle (*a*/Λ) changes for different wavelengths, Λ_Bragg_ = *λ*/(2*n*_B_), where the effective index *n*_B_ can be estimated from an approximation formula^24^,^25^


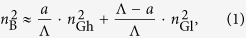


where *n*_Gh_ and *n*_Gl_ represent the refractive indices of high- and low-index regions of the grating, respectively. For ensuring the grating operating at the subwavelength regime, the chosen grating pitch of the SWG (Λ) must be substantially shorter than the Bragg period (Λ_Bragg_) so as to effectively suppress the diffraction effect^24^,^25^. On the other hand, however, the corresponding fabrication requirements about the complexity and cost should also be considered, implying that the SWG pitch width cannot be very small. Thus, we choose Λ to be 200 nm with a duty cycle of 50% under the following analysis, which can not only make the proposed PSR operating at the subwavelength regime in a broadband but also maximize the minimum feature size to alleviate the fabrication requirements.

### Device transmission analysis

**Figure 3:**
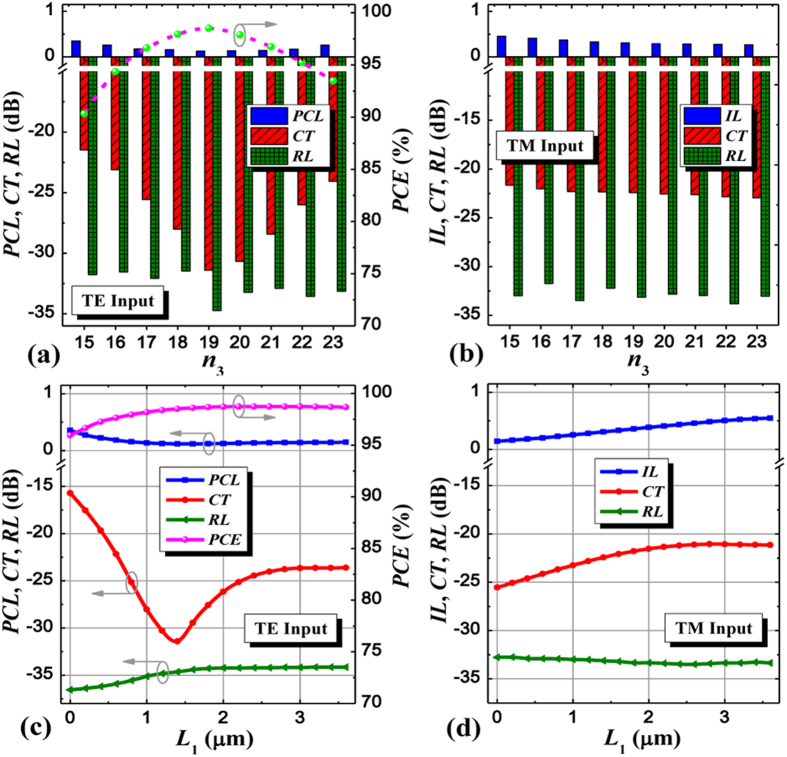
Device performance dependent on SWG multimode waveguide and a segment of strip waveguide. PCL, CT, RL, PCE, IL of the PSR as functions of the period number *n*_3_ (length: *n*_3_Λ) of the SWG multimode waveguide and the strip wavelength length *L*_1_ for (**a**,**c**) the input TE mode and (**b**,**d**) the input TM mode.

To evaluate the transmission characteristics and optimize the longitudinal sizes of the proposed PSR, simulations using a matured 3D finite-difference time-domain (FDTD) method are performed^37^, where PCL, CT, polarization conversion efficiency (PCE), IL, and reflection loss (RL) of the device are calculated and analyzed for both polarizations. Figure 3(a,b) show the device performance dependent on the SWG multimode waveguide length (*n*_3_Λ), where the input/output SWG-tapered transition is tapered from 0.45/0.35 μm to 1.35/0.05 μm in a period number of 10/6 and the SWG multimode width *w*_4_ is set to be 1.0 μm which can well separate the polarizations and reduce the CT. It is noteworthy that the input TE mode is more sensitive to *n*_3_ than the input TM mode because the input TE mode needs to be coupled to the adjacent partially-etched waveguide through SWG coupler, while the input TM mode directly transmits along the strip waveguide almost no “seeing” the SWG coupler. From these figures, the optimum length is located at *n*_3_ = 19 (*n*_3_Λ = 3.8 μm) corresponding to PCL = 0.12 dB, CT = −31.41 dB, RL = −34.74 dB, PCE = 98.52% for the input TE mode, and PCL = 0.31 dB, CT = −22.43 dB, RL = −33.13 dB for the input TM mode, respectively. Furthermore, to further separate both polarizations within the SWG multimode waveguide region, we add a segment of strip waveguide with a uniform width of 200 nm between the input and output tapered waveguides at the bar port, and its length *L*_1_ is studied in Fig. 3(c,d), where the output tapered waveguide length is *L*_2_ (=5.4 μm- *L*_1_). For the input TE mode, CT can reach the minimum value at the length of *L*_1_ = 1.4 μm, and PCE and PCL are tending to be better as *L*_1_ increases. In contrast, for the input TM mode, IL and CT gradually worsen when *L*_1_ increases from 0 to 3.0 μm. Therefore, we choose *L*_1_ to be 1.4 μm by making a tradeoff between both polarizations.

**Figure 4:**
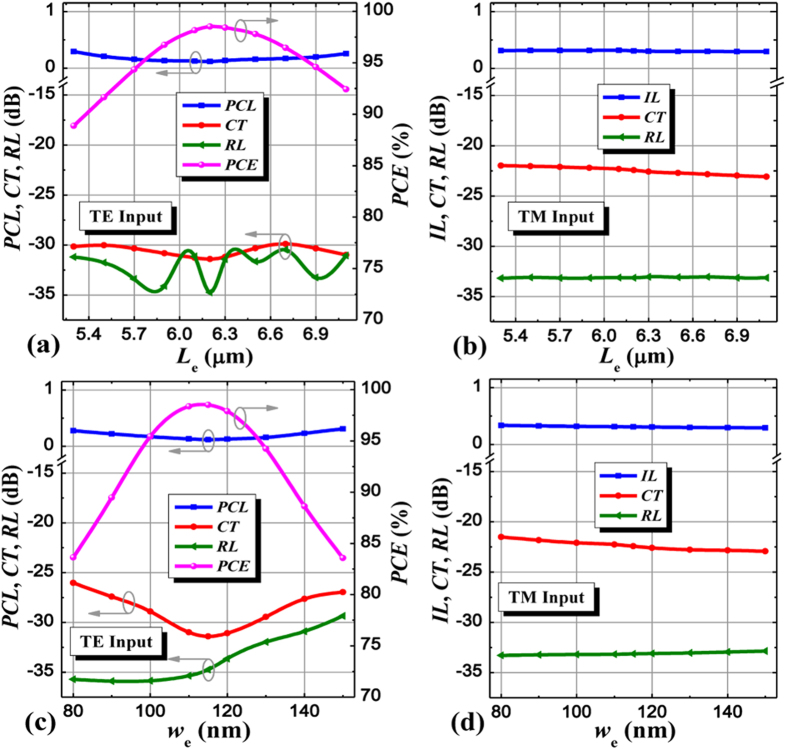
Device performance dependent on partially-etched waveguide. PCL, CT, RL, PCE, IL of the PSR as functions of the etching length *L*_e_ and width *w*_e_ of its partially-etched waveguide for (**a**,**c**) the input TE mode and (**b**,**d**) the input TM mode.

**Figure 5:**
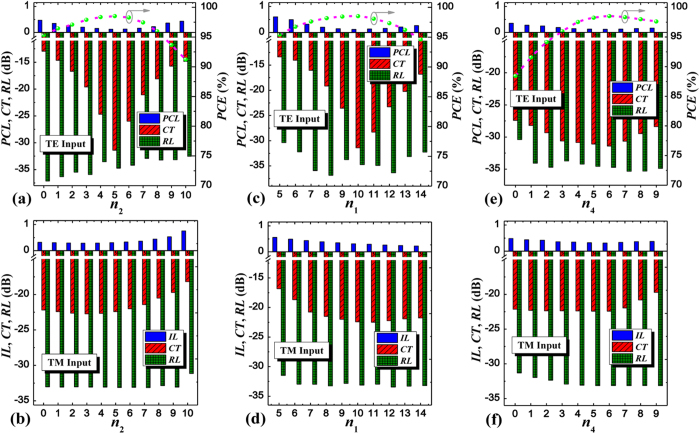
Device performance dependent on additional tapered waveguide and SWG-tapered transitions. PCL, CT, RL, PCE, IL of the PSR as functions of period numbers of the additional tapered waveguide *n*_2_ (length: *n*_2_Λ), input SWG-tapered transition *n*_1_ (length: *n*_1_Λ), and output SWG-tapered transition *n*_4_ (length: *n*_4_Λ) for (**a**,**c**,**e**) the input TE mode and (**b**,**d**,**f**) the input TM mode.

For effectively converting the coupled TE mode to output TM mode at the cross port, we introduce a partially-etched waveguide and its etching length *L*_e_ and width *w*_e_ are investigated^38^, as illustrated in Fig. 4. For the propagation of TM mode, it only transmits along the bottom strip waveguide almost neglecting the SWG coupler and even the partially-etched waveguide on the other side, thus the performance of TM mode is little affected by *L*_e_ and *w*_e_. From Fig. 4, variation of the etching width *w*_e_ has a more effect on the device performance than that of the etching length *L*_e_, leading to a more tight fabrication tolerance for *w*_e_. If PCE > 96% should be kept for the input TE mode, *L*_e_ and *w*_e_ must be controlled within the ranges of [5.9, 6.7] μm and [104, 125] nm, respectively, around their optimum values of *L*_e_ = 6.2 μm and *w*_e_ = 115 nm. Meanwhile, the corresponding PCL, CT, and RL are lower than 0.18 dB, −29 dB, and −30 dB, respectively, within these ranges. While, for the input TM mode, the device performance is nearly unchanged within the calculated ranges for both parameters due to its quite weak coupling with the adjacent partially-etched waveguide. Moreover, to enhance the device performance, we add a tapered waveguide extended from the partially-etched waveguide in the lateral end of the input SWG-tapered transition, which is employed to efficiently pull the input TE mode to the upper high index region and not affect the propagation behavior of input TM mode. Thus its length (*n*_2_Λ) takes an important role in the design process. If this length is too short, the added advantage is quite weak without significant improvements, also this length cannot be very long since the propagation of input TM mode will deteriorate if it reaches the main distribution region of input TM mode, incurring the increase of CT, the behaviors of which have also been quantitatively analyzed, as shown in Fig. 5(a,b), which agrees well with our analysis. From Fig. 5(a,b), we find an optimum value of *n*_2_ = 5, which is dominated by input TE mode since the performance of input TM mode changes slightly as *n*_2_ < 6. If *n*_2_ further increases, however, not only the performance of input TE mode but also that of input TM mode will degenerate because the added waveguide is gradually close to the main distribution of input TM mode, thus unwanted mode coupling will take place inevitably. In addition, compared with no such structure (*n*_2_ = 0), the CT and PCL of input TE mode are reduced by ~18 dB and ~0.35 dB, respectively, which also demonstrates the great advantage of the additional tapered waveguide in the PSR and this optimized structure has already been employed in the aforementioned analysis. The device performance dependent on the lengths of input and output SWG-tapered transitions (*n*_1_, *n*_4_) is also investigated in Fig. 5(c–f). It is seen that the output transition shows relatively low performance sensitivity than the input one except for the PCE because the output transition is more close to the polarization conversion region thus its influence may be stronger. To guarantee CT <−20 dB for both polarizations, *n*_1_ can be varied from 9 to 13 whereas *n*_4_ can almost be chosen arbitrary value within the range from 0 to 9. If both considering the criteria of PCE > 96%, however, the range of variation for *n*_4_ is from 3 to 9 instead.

### Device spectral response and fabrication tolerance

**Figure 6:**
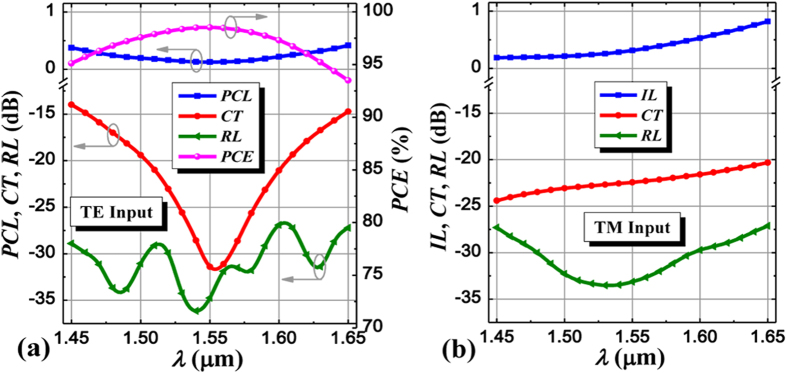
Device spectral response. Wavelength dependence of the PSR performance for (**a**) the input TE mode and (**b**) the input TM mode.

**Figure 7:**
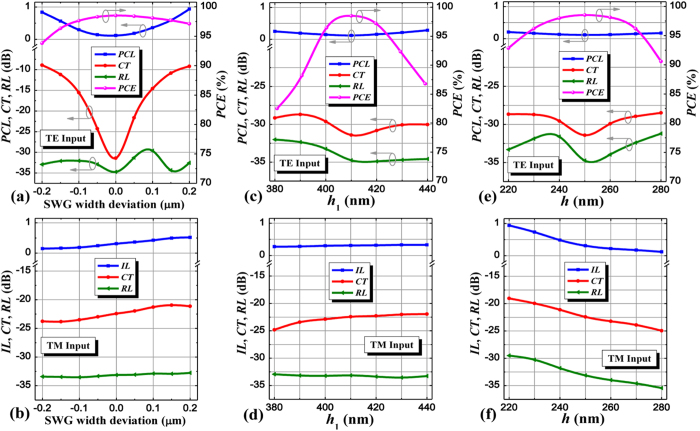
Fabrication tolerance. PCL, CT, RL, PCE, IL of the PSR as functions of the whole SWG width deviation and wavelength height *h*_1_, *h* for (**a**,**c**,**e**) the input TE mode and (**b**,**d**,**f**) the input TM mode.

Figure 6 presents the wavelength dependence of the proposed PSR, where the wavelength is ranged from 1.45 to 1.65 μm, covering the whole S, C, L bands and a part of U band, and material dispersions of Si and SiO_2_ are not considered due to their quite low dispersions within this range. PCL, CT, and PCE of the input TE mode can achieve the optimum values nearly at the central wavelength of 1.55 μm, while IL and CT of the input TM mode gradually worsen with the increase of wavelength. If CT <−20 dB is required for both polarizations, the allowable bandwidth is ~104 nm from 1.502 to 1.606 μm, which is mainly restricted by input TE mode because the CT of input TM mode is always under −20 dB within the whole wavelength range. Meanwhile, PCL, RL, and PCE of the input TE mode are lower than 0.25 dB, −27 dB, and higher than 96.7%, respectively; IL and RL of the input TM mode are lower than 0.58 dB and −29 dB, respectively, within this bandwidth. Moreover, we also study the fabrication tolerance to key structural parameters of the PSR for both polarizations, such as the whole SWG width deviation and the waveguide thicknesses (*h*_1_, *h*) variation, as shown in Fig. 7. To keep CT <−20 dB, the whole SWG width deviation must be controlled from −70 nm to 60 nm, which requires the etching process in a relatively high precision. For the waveguide thickness variation, we employ PCE to characterize the device performance since thickness variations affect the PCE more obviously and directly than the CT. To keep PCE > 96%, the waveguide thicknesses *h*_1_ and *h* must be controlled within the ranges of [398, 423] nm and [228, 271] nm, respectively, and the corresponding etching depth *h*_e_ is within the range of [139, 182] nm (see Figure S1 in the Supplement Information). It is noted that, from these figures, the performance of input TM mode clearly degenerates as *h* reduces or *h*_e_ increases, and this is because the TM mode will tend to the cladding layer with increased loss under this condition. For the SWG pitch width Λ, a key parameter in our design, it is fixed to 200 nm within the design process and its size deviation due to fabrication errors can be under ten nanometers at present^39^,^40^, thus the operation regime for the SWG and the device performance will not be affected significantly. The device performance dependent on the duty cycle *a*/Λ of SWG structure is also analyzed (see Figure S2 in the Supplement Information). For keeping CT <−20 dB, the duty cycle must be controlled from 46.5% to 55.5%, corresponding to the high index segment width of SWG varying from 93 to 111 nm, which can be readily realized using the present fabrication technology^39^,^40^. To fabricate the proposed PSR, a SOI wafer with 410 nm thick top silicon layer is employed. Firstly, the whole PSR structure is patterned with a deep ultraviolet lithography or an electron-beam lithography process and followed by an inductively coupled plasma etching process to form the whole PSR on the top silicon layer with a uniform thickness of 410 nm. Then, a second lithography and etching processes are required to form the bottom structure with a thickness of 250 nm including the input/bar port, SWG multimode waveguide region, input/output SWG-tapered transition, and partially-etched waveguide region^12^,^41^. Finally, the chip is covered with SiO_2_ layer using a plasma enhanced chemical vapor deposition or other film deposition processes^41^. Additionally, chemical mechanical polishing should also be used to planarize the chip surface^42^.

**Figure 8:**
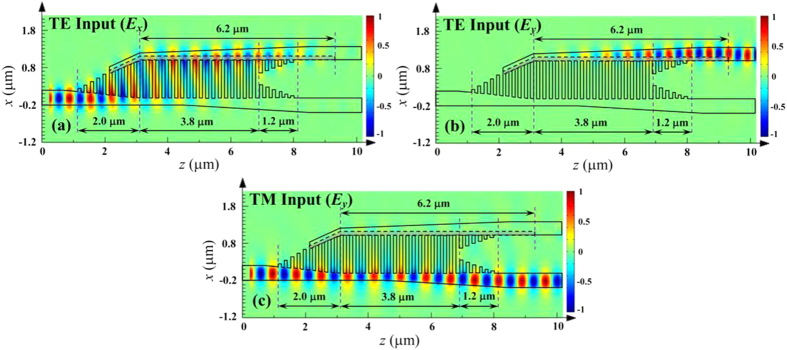
Field evolution. Field evolution of (**a**) the *E*_*x*_ and (**b**) *E*_*y*_ components of the input TE mode, and (**c**) *E*_*y*_ component of the input TM mode along the propagation direction through the designed PSR, where the lengths of the input/output SWG-tapered transition, SWG multimode waveguide region, and partially-etched waveguide are 2.0/1.2 μm, 3.8 μm, and 6.2 μm, respectively.

The field evolution of input TE and TM modes along the propagation distance of the proposed PSR is also performed using the 3D-FDTD method, as plotted in Fig. 8, where *n*_1_ = 10, *n*_2_ = 5, *n*_3_ = 19, *n*_4_ = 6, *w*_1_ = 0.4 μm, *w*_2_ = 0.45 μm, *w*_3_ = 0.2 μm, *w*_4_ = 1.0 μm, *w*_5_ = 1.2 μm, *w*_6_ = 0.15 μm, *w*_7_ = 0.35 μm, *w*_8_ = 0.05 μm, *w*_9_ = 0.35 μm, *L*_1_ = 1.4 μm, *L*_2_ = 4.0 μm, *L*_e_ = 6.2 μm, *w*_e_ = 115 nm, *h* = 250 nm, *h*_1_ = 410 nm, *a* = 100 nm, Λ = 200 nm, and *λ* = 1.55 μm. For the input TE mode, it gradually couples to the adjacent partially-etched waveguide and converts to the output TM mode simultaneously at the cross port within a device length of only 8.2 μm. As to the input TM mode, it always transmits along the input strip waveguide and directly outputs from the bar port nearly without influence of the partially-etched SWG coupler. As a consequence, at the output ports, we can get two separated modes with the same polarization state, corresponding to the realization of an ultracompact and high efficient PSR based on a partially-etched SWG coupler.

## Discussion

We have proposed an ultracompact and high efficient silicon-based PSR using a partially-etched SWG coupler between the input and output waveguides, where SWG-tapered transitions are employed at both ends to increase the coupling efficiency. Using the methods of full-vectorial FDFD and matured 3D-FDTD, the SWG coupler and its lateral partially-etched waveguide including SWG-tapered transitions have been analyzed in detail to make the input TE mode convert to the output TM mode from the cross port while the input TM mode is directly output from the bar port almost without coupling, and the whole device performance has been systematically studied. In the present PSR, the input TE mode is coupling to the adjacent partially-etched waveguide and converting to the output TM mode simultaneously without some intermediate modes as a bridge, leading to a great reduction in length. Moreover, we also introduce an additional tapered waveguide extended from the partially-etched waveguide in the lateral end of the input SWG-tapered transition to further improve the device performance. Results show that the proposed PSR having a total device length as short as 8.2 μm is achieved, which is the shortest one reported until now. The corresponding PCL, CT, RL, and PCE for the input TE mode are 0.12 dB, −31.41 dB, −34.74 dB, and 98.52%, IL, CT, and RL for the input TM mode are 0.31 dB, −22.43 dB, and −33.13 dB, respectively, at wavelength of 1.55 μm, which are also relatively well. Besides, the bandwidth can be enlarged to ~104 nm (from 1.502 to 1.606 μm) for keeping CT <−20 dB and fabrication tolerances to the key structural parameters have also been investigated. Such ultracompact PSR with characteristics of low loss, low crosstalk, large bandwidth, and relatively easy fabrication, has promising applications for realizing on-chip compact polarization diversity schemes, constructing polarization-independent PICs or quantum photonic circuits, and also building on-chip polarization-division-multiplexing transmission systems. Moreover, the suggested PSR could be applied to other material platforms or operating wavelengths.

## Methods

In this study, two numerical methods (full-vectorial FDFD and 3D-FDTD) are combined together to perform the modal and transmission analyses of our proposed PSR.

### Waveguide modal analysis

The modal characteristics of silicon-based strip waveguide are investigated using an accurate full-vectorial FDFD method due to its high index structure. For this method, it is originally employed to study the modal features of bending waveguides^36^. If the bending radius *R* approaches towards the infinity, however, the equations for bending waveguides will be reduced to those for straight ones, showing more general and versatile. Using a two-dimensional Yee’s mesh and the central finite difference scheme, the Maxwell’s curl equations can be described as


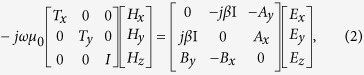



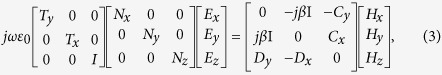


where *E*_*x*_, *E*_*y*_, and *E*_*z*_ are electric components, *H*_*x*_, *H*_*y*_, and *H*_*z*_ are magnetic components, all depending on *x* and *y* coordinates, *N*_*x*_, *N*_*y*_, and *N*_*z*_ are the diagonal matrices, *I* is the identity matrix, and *β* is the propagation constant. The matrices of *T*_*x*_ and *T*_*y*_ will turn into the identity matrixes due to the radius *R* tending to the infinity for the case of straight waveguide. Other definitions for operators *A*_*x*_*E*_*z*_, *A*_*y*_*E*_*z*_, *B*_*y*_*E*_*x*_, *B*_*x*_*E*_*y*_, *C*_*x*_*H*_*z*_, *C*_*y*_*H*_*z*_, *D*_*x*_*H*_*y*_, and *D*_*y*_*H*_*x*_ can be found in Ref. 36. To further improve the calculation efficiency, the eigenvalue matrix equation in terms of transverse electric field components, *E*_*x*_ and *E*_*y*_, can be derived as


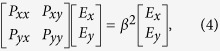


where operators *P*_*xx*_, *P*_*xy*_, *P*_*yx*_, and *P*_*yy*_ can be found in ref. 36. Within the calculation of guided-modes, the waveguide structure is surrounded by perfectly matched layers (PMLs) to effectively eliminate the non-physical reflections from edges of the computational window and the convergence is also tested by fining the meshes around the high index region and its corners. Based on this method, we can choose the suitable strip waveguide widths (*w*_1_, *w*_3_) to efficiently make the input polarizations separate within the region of the input SWG-tapered transition.

### Device transmission analysis

A matured 3D-FDTD method^37^, formulas of which are directly derived from Maxwell’s equations with the help of 3D Yee’s mesh, is used to study the transmission features of the suggested PSR including structural optimizations. Owing to the same computational window and mesh grid in the transverse direction with those of the FDFD method, the mode fields calculated by the FDFD method can be easily and directly incorporated into the 3D-FDTD method as its excitation source for the transmission analysis. Meanwhile, PMLs are also imposed at the edges of the computational window. Using this method, we analyze the device performance and optimize its structural dimensions in detail including tolerance analysis to achieve an ultracompact and high efficient PSR.

Within the transmission analysis, we employ PCL, CT, RL, and PCE to characterize the device performance for the input TE mode, and their definitions are expressed as


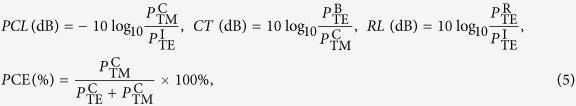


where *P*_*y*_^*x*^ stands for the power at the *x* port (I: input port, B: bar port, C: cross port, R: reflection port) with *y* polarization state (TE: TE polarization, TM: TM polarization). For the input TM mode, we use IL, CT, and RL instead, and their definitions are also expressed as


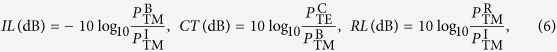


where the definition of *P*_*y*_^*x*^ is same with that of the input TE mode.

## Additional Information

**How to cite this article**: Xu, Y. and Xiao, J. Ultracompact and high efficient silicon-based polarization splitter-rotator using a partially-etched subwavelength grating coupler. *Sci. Rep*. **6**, 27949; doi: 10.1038/srep27949 (2016).

## Supplementary Material

Supplementary Information
