# Supplementary material for: Ultracompact and high efficient silicon-based polarization splitter-rotator using a partially-etched subwavelength grating coupler
Source: Sci Rep. 2016 Jun 16;6:27949. doi: 10.1038/srep27949 (PMC4910076; doi:10.1038/srep27949)
Supplement: Supplementary Information [file srep27949-s1.pdf]

Supplementary Information

Ultracompact and high efficient silicon-based polarization splitter-rotator using a partially-etched subwavelength grating coupler

Yin Xu and Jinbiao Xiao*

National Research Center for Optical Sensing/Communications Integrated Networking, School of Electronic Science and Engineering, Southeast University, Nanjing 210096, China
**Corresponding author, e-mail address: jbxiao@seu.edu.cn*

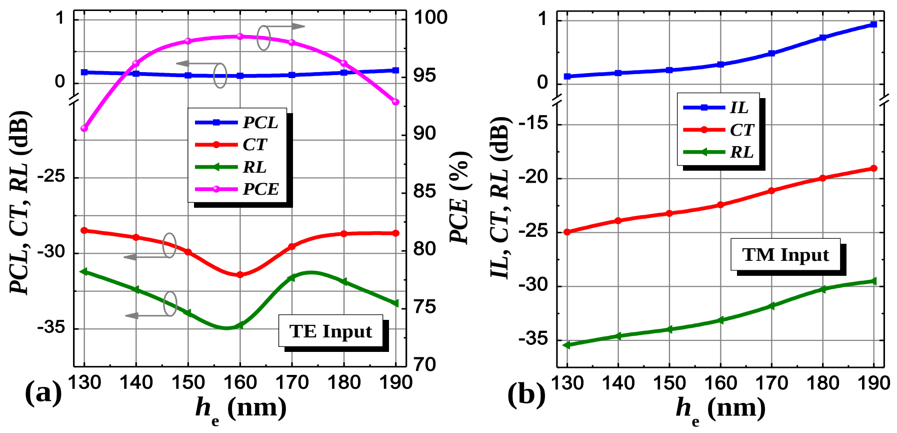

Supplementary Figure S1. PCL, CT, RL, PCE, IL of the PSR as a function of the waveguide etching depth h_e for (a) the input TE mode and (b) the input TM mode.

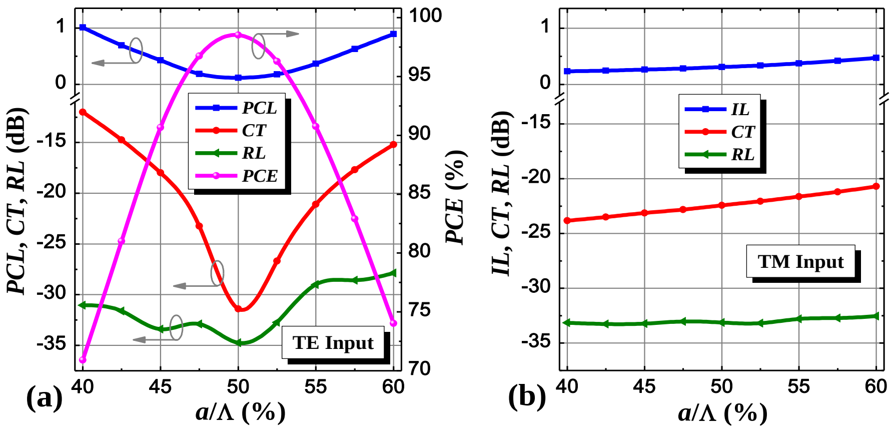

Supplementary Figure S2. PCL, CT, RL, PCE, IL of the PSR as a function of the duty cycle *a*/Λ of subwavelength grating structure for (a) the input TE mode and (b) the input TM mode.
